# Comparison of tunnel and VISTA techniques for multiple gingival recession treatment: A systematic literature review

**DOI:** 10.34172/japid.2024.007

**Published:** 2024-04-23

**Authors:** Ksenija Matvijenko, Rokas Borusevičius

**Affiliations:** ^1^Lithunian University of Health Sciences, Medical Academy, Faculty of Odontology, Kaunas, Lithuania; ^2^Vilnius University, Faculty of Medicine, Institute of Odontology, Vilnius, Lithuania

**Keywords:** Gingival recessions, Systematic review, Tunnel technique, VISTA

## Abstract

**Background.:**

Gingival recession (GR) has become one of the most common concerns in oral mucosal diseases. It causes discomfort such as root hypersensitivity, root caries, and aesthetic problems, leading to the development of various surgical techniques to address GRs. This study compared the non-advanced tunnel and m-VISTA techniques in treating multiple GRs.

**Methods.:**

A literature search related to the efficiency of non-advanced tunnel and m-VISTA techniques was conducted in MEDLINE (PubMed), EMBASE (ScienceDirect), Cochrane Central Register of Controlled Trials (Cochrane Library), Springer Link, and Google Scholar. Randomized controlled trials (RCTs) reporting periodontal parameters published in the recent four years (2019–2023) were included and assessed for the risk of bias. All in vitro, animal, pilot studies, case reports, and case series were excluded.

**Results.:**

Five RCTs were included with 195 cases of GRs. Comparing the two techniques revealed a significant increase in keratinized tissue width (KTW) from baseline to 6 months (-1.4 mm), in clinical attachment level (CAL) (-2.65 mm), and in recession depth (-2.7 mm) for the tunnel technique. On the other hand, a significant increase in GR width (-2.26 mm) was found in the m-VISTA group. Finally, there were no significant differences in probing depths.

**Conclusion.:**

Both techniques were effective in root coverage and may be valuable for treating multiple GRs.

## Introduction

 Gingival recession (GR) is one of the most prevalent oral mucosal diseases.^1^ GR is clinically described by apical migration of gingival tissues, resulting in root surface exposure.^1^ GRs can manifest as localized or generalized, affecting one or more surfaces. GR has been linked to the aging process for decades, but the evidence supporting this association remains unclear.^2^ While aging may increase the likelihood of GR, it is not an inevitable consequence.^2^

 The pathophysiology of GR involves both direct causes and predisposing factors. Predisposing factors include dehiscences, fenestrations, reduced alveolar bone ridge thickness combined with the thin gingival biotype, and labial frenum attachment.^3^ Direct causes encompass chronic trauma, chronic periodontal inflammation, periodontal treatment, and occlusal trauma.^3^

 In 1985, Miller proposed a widely used classification system for marginal tissue recessions based on the gingival margin’s level concerning the mucogingival junction and the underlying alveolar bone.^4^ In 2010, Mahajan modified Miller’s classification into four classes.^5^ In addition, it distinguished among three GR types concerning the amount of interdental clinical attachment loss, as proposed by Cairo et al^4^ in 2011.Nevertheless, Miller’s classification is still the most widely used among all the classification systems.^6^

 However, GR can elicit patient concerns, such as root hypersensitivity, erosion, root caries, and aesthetic issues.^7^ The increasing emphasis on aesthetics has prompted the development of various surgical procedures to cover exposed roots.^8^

 Today, there are several known treatments for GR. Although the coronally advanced flap has been the most commonly used method to treat multiple GRs, new, less invasive methods have been proposed, such as the modified vestibular incisional approach to the periosteal tunnel (m-VISTA).^9^ The m-VISTA technique involves a vertical vestibular incision, typically at the jaw frenum level, followed by the elevation of a subperiosteal tunnel through the incision that should include the gingival margin of at least one tooth adjacent to the teeth requiring GR treatment.^10^ Although this technique was initially designed to treat Miller Class I and II recessions in the maxilla, it can be used in other areas (including more than two recessions) as well.^11^

 Moreover, another treatment for GR is the standard non-advanced tunnel technique (TT), a minimally invasive procedure without vertical incisions that preserves the interdental papilla.^11^ Proposed by Zabalegui and later modified over the years, the TT has recently gained popularity among clinicians due to its promising clinical and aesthetic outcomes in treating GR defects.^12^ However, the evidence for the efficacy of TT is controversial.^13^

 This literature review aimed to compare the two techniques, i.e., to investigate the efficacy in the treatment of multiple GRs.

## Methods

###  Protocol and questions for the systematic review

 The question for this systematic literature review was formulated based on the PICOS model (P, Patient/Problem/Population; I, Intervention/Indicator; C, Comparison; O, Outcome of interest; S, Study designs) described in the Cochrane Handbook for Systematic Reviews of Interventions 5.1.0: which technique is more accurate in the coverage of multiple GRs? In this literature review, P: patients with two or more RT1 (Miller I, Miller II) and/or RT2 (Miller III) GRs, I: GR closure/treatment with the tunnel or m-VISTA techniques, C: differences in periodontal parameters before and after treatment of recessions, O: periodontal parameters: probing depth (PD), clinical attachment level (CAL), gingival recession depth (RD), width of the keratinized tissue (KTW), width of the gingival recession (GRW), S: randomized controlled clinical trials (RCTs).

###  Search methods

 This systematic review of the scientific literature has been prepared in accordance with the PRISMA (Preferred Reporting Item for Systematic Reviews and Meta-Analyses) requirements. The articles were searched by one independent researcher (KM).^14^

###  Inclusion criteria

- Publication type: RCTs
- A study sample of at least 10 patients
- Follow-up period ≥ 6 months
- The study should clearly state the outcomes (PD, CAL, RD, KTW, GRW), and the statistical significance of the difference in the change between baseline and after 6 months
- Papers written in English
- Articles relevant to the topic

###  Exclusion criteria

- Literature reviews or meta-analyses, single case studies, lectures, and letters
- Articles investigating the modified TT
- Articles investigating localized, isolated GRs
- Publications that do not provide sufficient information for the study
- Publications older than 10 years
- Articles written in a language other than English

###  Sources of information

 For the systematic review of the scientific literature, articles were searched in the electronic databases MEDLINE (PubMed), EMBASE (ScienceDirect), Cochrane Central Register of Controlled Trials (Cochrane Library), Springer Link, and Google Scholar. A structured search of these databases was performed without time or other limitations to answer the question - which technique is more effective in the treatment of multiple GRs?

###  Electronic data search strategy

 The selection of articles was started on August 11, 2023. The last search was performed on November 13, 2023. Scientific publications were retrieved by entering keywords and combinations of keywords found in the term database: “tunnel technique,” “VISTA,” “gingival recessions,” “treatment,” and “recession coverage.”

###  Article selection process

 The articles were selected in several steps to avoid errors, such as excluding eligible articles and exclusion from the systematic literature review. The first step was the selection of publications according to the title (articles had to be written in English and not more than 10 years old), followed by an examination of the abstracts of the selected publications according to the criteria listed below. The abstracts were read, and those not meeting the selection criteria were rejected. In the final stage, the full-text articles were read, and after assessing their eligibility for the systematic review, the articles were selected for inclusion in this systematic literature review.

###  Quality assessment

 The risk of bias in the selected studies was assessed using the Cochrane risk of bias tool (RoB 2.0). Five domains were assessed: the randomization process, deviations from the intended interventions, missing outcome data, the outcome, and the selection of reported results. All the domains were categorized as low, unclear, or high risk of bias. “Low risk of bias” was assigned when a low risk of bias was identified in all domains, or “some concerns” when at least one domain was assessed as posing some concerns but was not at a high risk of bias in any individual domain.

###  Process for extracting data from articles

 The research data selected for the systematic literature review were collected and tabulated according to the Cochrane Training methodological guidelines. The following data were extracted from the studies:

- General information: main author of the study and year
- Type of study
- Study sample (number of patients)
- Study methodology (study blinding, randomization, and allocation concealment)
- Statistical analysis, criteria, measurement parameters, tests applied
- Study results and conclusions

## Results

###  Study selection

**Figure 1:**
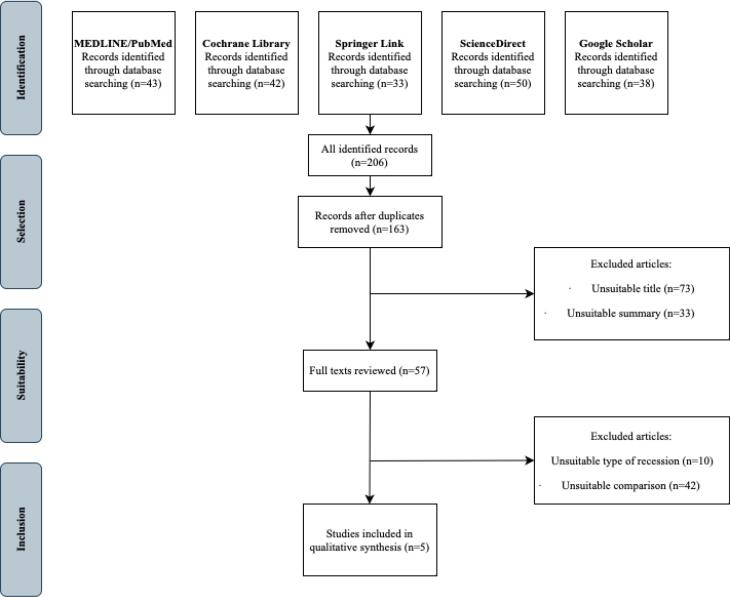
The review search and selection flowchart

 The initial search identified 206 articles. The selection strategy is illustrated in the PRISMA diagram (Figure 1). After eliminating duplicates, 163 articles were screened. After evaluating titles and abstracts, 57 articles were selected for full-text reading, and ultimately, five articles were deemed eligible for inclusion in this systematic review.

###  Quality assessment

**Figure 2:**
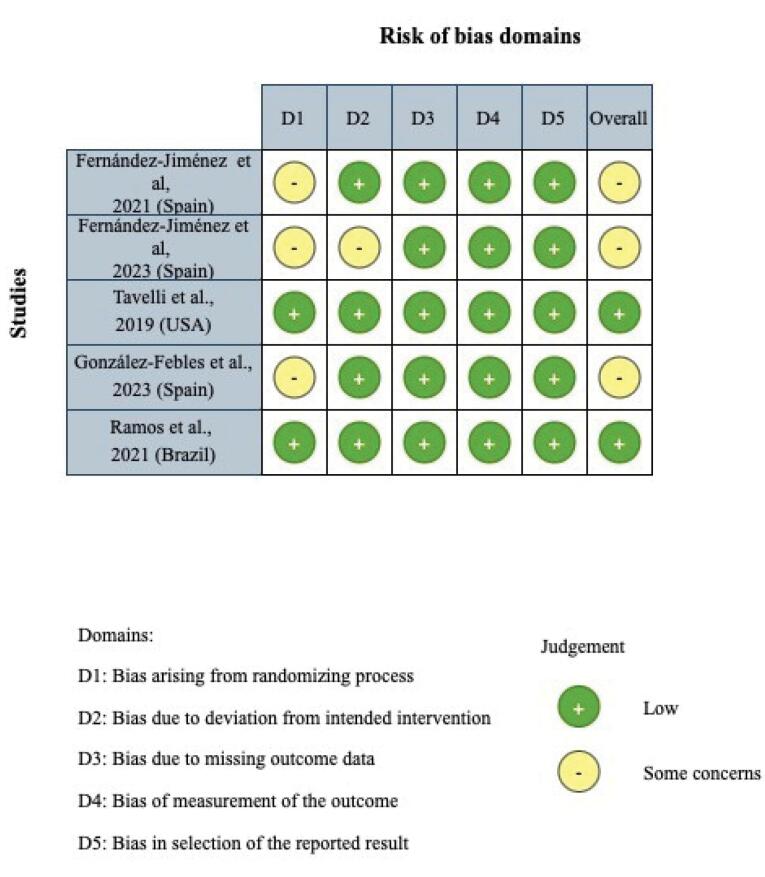
Risk of bias assessment using the modified RoB 2.0 tool

 The risk of bias in two studies was evaluated as low, while it raised some concern in the other three studies. Detailed results regarding the risk of bias for the included studies are depicted in Figure 2.

###  Study characteristics

 The primary characteristics of the articles included in this review are provided in Table 1, with a summary of detailed results in Table 2. All included articles were RCTs published between 2019 and 2023. These studies analyzed both periodontal parameters and subjective parameters. However, only one of the studies reported results from subjective outcomes, which were assessed using the standard visual analog scale (VAS). The number of participants varied from 10 to 20, with ages ranging from 18 to 73 years. Recessions were treated in both jaws, totaling 197 cases, and the follow-up period ranged from 6 months to 12 years. However, this study focused specifically on the results after 6 months (excluding the 12-month and 12-year results from the comparison).

**Table 1:** Studies characteristics

**Studies**	**Study design**	**Evaluation parameters**	**No. of** **participants**	**Age (y)**	**No. of treated recessions**	**Site of recession**
Fernández-Jiménez et al,^16^ 2021 (Spain)	RCT	Periodontal, subjective	10	41-61	38	Both jaws
Fernández-Jiménez et al,^15^ 2023 (Spain)	RCT	Periodontal, subjective	12	31-73	44	Both jaws
Tavelli et al,^18^ 2019 (USA)	RCT	Periodontal, subjective	12	≥ 18 years	34	-
González-Febles et al,^19^ 2023 (Spain)	RCT	Periodontal, subjective	15	≥ 18 years	41	Both jaws
Ramos et al,^17^ 2021 (Brazil)	RCT (split-mouth)	Periodontal, subjective	19	18 - 59	38	Both jaws

RCT: randomized controlled clinical trial

**Table 2:** Studies results

**Studies**	**Recession Class**	**Procedure**	**Periodontal parameters at baseline and after 6 months (SD)**					**Change Base-line-6 months(SD)**				
			**PD** **(mm)**	**CAL ** **(mm)**	**RD** **(mm)**	**KTW (mm)**	**GRW (mm)**	**PD ** **(mm)**	**CAL (mm)**	**RD ** **(mm)**	**KTW (mm)**	**GRW (mm)**
Fernández-Jiménez et al,^16^ 2021 (Spain)	Miller III	m-VISTA	1.80 (0.52)	4.92 (1.29)	3.12 (0.89)	2.63 (1.22)	4.37 (1.13)	0.09 (0.15)	-1.76 (1.07)	-1.85 (0.92)	1.11 (1.04)	-2.26 (1.25)
			1.89 (0.67)	3.16 (1.36)	1.27 (1.9)	3.74 (2.26)	2.11 (2.38)					
Fernández-Jiménez et al,^15^ 2023 (Spain)	Miller III	m-VISTA	1.75 (0.45)	4.6 (1.01)	2.85 (0.72)	2.71 (1.05)	4.08 (1.06)	-0.04 (0.51)	-1.84 (1.07)	-1.73 (0.56)	0.85 (1.19)	-2.17 (1.25)
			1.75 (0.43)	2.87 (0.76)	1.12 (0.74)	3.57 (1.62)	1.94 (1.27)					
Tavelli et al,^18^ 2019 (USA)	RT1	TT	0.93 (0.41)	3.22 (1.02)	2.29 (0.96)	2.54 (1.16)	-	0.36 (0.08)	-1.63 (0.35)	-1.98 (0.39)	-0.52 (0.47)	-
			1.29 (0.49)	1.59 (0.67)	0.31 (0.57)	2.01 (0.69)	-					
González-Febles et al,^19^ 2023 (Spain)	RT1/RT2	TT	1.8 (0.6)	4.6 (2.4)	2.8 (1.8)	2.3 (1.3)	-	0.05 (0.6)	-2.65 (2.4)	-2.7 (1.8)	-1.4 (1.4)	-
			1.85 (0.05)	1.95 (0.1)	0.1 (0.05)	0.9 (0.1)	-					
Ramos et al,^17^ 2021 (Brazil)	RT1	TT	1.65 (0.49)	5.36 (1.44)	3.71 (0.95)	2.48 (1.34)	4.53 (0.81)	0.00 (0.1)	-0.8 (0.42)	-1.87 (0.04)	0.95 (0.08)	-1.19 (0.38)
			1.65 (0.59)	3.49 (1.5)	1.84 (0.91)	3.43 (1.26)	3.34 (1.19)					

TT: tunnel technique, SD: standard deviation, PD: probing depth, CAL: clinical attachment level, RD: gingival recession depth, KTW: width of the keratinized tissue, GRW: width of the gingival recession.

## Discussion

 Since several systematic reviews have already assessed the predictability of root coverage procedures, evidence regarding the efficacy of the TT and m-VISTA is not yet conclusive. This literature analysis evaluated periodontal parameters for (non-advanced) tunnel and m-VISTA technique outcomes. Additionally, subjective parameters such as postoperative pain and aesthetic outcomes were considered. While discomfort, pain, and aesthetic outcomes are subjective and challenging to assess, they are crucial patient parameters.^20^

 The aesthetic score (AS) was used to evaluate the subjective parameters, and patients’ perception of acute pain after surgery was recorded using a pain diary developed by UPV/EHU.^15^ The maximum pain intensity felt was measured on a VAS ranging from 0 to 100 mm. Furthermore, patients’ perception of the aesthetic outcome was assessed six months after surgery on a VAS scale ranging from no aesthetic outcome (VAS = 0) to the most likely aesthetic outcome (VAS = 100). Fernández-Jiménez et al^16^ reported that the mean VAS intensity of pain experienced was 13.51 ± 12.86. After the first day post-operatively, nearly half (four) of the patients had no pain, and the mean VAS score was 81.90 ± 17.30.

 Regarding periodontal parameters, the TT was effective in treating both GR RT1 (Miller I, Miller II) and RT2 (Miller III) classes.^17-19^ The most significant changes in periodontal parameters were observed: 0.00 mm in probing depth, -2.65 mm in CAL, -2.7 mm in recession depth, and -1.4 mm in KTW. TT is designed to treat multiple and large GRs, often found in challenging areas for root coverage.^21^ It has been suggested that improved aesthetic outcomes, faster healing, and reduced patient morbidity are among the main advantages of TT. Additionally, the TT helps maintain adequate and continuous blood supply for excellent graft adaptation in the recipient area.^22^

 Travelli et al^18^ suggested that TT was a highly effective procedure in treating GR defects, exhibiting an overall mean root coverage of 82.8% for single and 87.9% for multiple GR defects and a complete root coverage of 47.2% and 57.5% for single and multiple GR defects, respectively. TUN was more effective in treating maxillary and Miller Class I and II GR defects.

 On the other hand, both trials analyzing the m-VISTA technique focused on treating the GR Miller Class III defects.^15,16^ The most significant changes in periodontal parameters were observed: -0.04 mm in probing depth, -0.84 mm in CAL, -0.85 mm in recession depth, 0.85 mm in KTW, and -2.26 in GRW. On the other hand, Alkababji et al^23^ claimed in their split-mouth randomized clinical trial that multiple Miller Class I and Class II recessions in the maxilla can be effectively treated with the VISTA technique. This technique avoids incisions or trauma to the marginal gingival tissues to preserve the vascularization of the treated area. In addition, it involves stabilization of the gingival margins, referred to as coronally anchored suturing, to promote healing by preventing micromotion, a major obstacle in the healing process.^16^

 Comparing both techniques, a significant increase was noticed in KTW from baseline to 6 months (-1.4 mm), in CAL (-2.65 mm), and in recession depth (-2.7 mm) by TT. On the other hand, a significant increase in GRW (-2.26 mm) was found in the m-VISTA group. Finally, there were no significant differences in probing depth.

 However, while the results of this literature review are informative, the lack of homogeneity in this study is a major limitation when comparing both techniques. Statistical heterogeneity was estimated using χ^2^ (*Q* value) and *I*^2^ analyses. A χ^2^ *P* value of > 0.50 and an *I*^2^ value of 55% were interpreted as moderate heterogeneity. Furthermore, the small number of randomized clinical trials and differences in surgical protocols or assessments between studies limit data comparison. Although these differences may be partly attributed to methodological issues (partial recording protocols, convenience samples), it is reasonable to infer that they may also be explained by different age ranges of the cohorts, periodontal profiles, possible ethnic/genetic determinants, oral hygiene habits, and exposure to risk factors. Nevertheless, more clinical trials with a longer follow-up period are needed to arrive at a concrete conclusion about their advantages and evaluate these techniques more accurately.

## Conclusion

 Based on the findings of this review, it can be concluded that both methods (m-VISTA and TTs) are effective procedures for treating multiple gingival recessions of RT1 (Miller I and Miller II) and RT2 (Miller III) classes. While the TT technique may yield superior results in terms of KTW, CAL, and recession depth, m-VISTA provides a decrease in gingival recession width.

## Competing Interests

 The authors declare that they have no competing interests.

## Data Availability Statement

 The data are available upon request from the corresponding author.

## Ethical Approval

 Not applicable.

## Funding

 None.
